# Optimizing Light Intensity for the Co-Production of Fucoxanthin and Polyunsaturated Fatty Acids in *Isochrysis galbana*

**DOI:** 10.3390/biotech15020041

**Published:** 2026-06-01

**Authors:** David Kwame Amenorfenyo, Wenquan Zheng, Zhe Cao, Junhao Huang, Zitong Deng, Jiacheng Ruan, Feng Li, Hua Xiao

**Affiliations:** 1 College of Fisheries, Guangdong Ocean University, Zhanjiang 524088, China; davidamenorfenyo@yahoo.com (D.K.A.); 11135432oo@stu.gdou.edu.cn (W.Z.); caozh672@stu.gdou.edu.cn (Z.C.); 11135410jj@stu.gdou.edu.cn (J.H.); 11135405bb@stu.gdou.edu.cn (Z.D.); 2 School of Electronic and Information Engineering, Guangdong Ocean University, Zhanjiang 524088, China; ruanjiacheng@stu.gdou.edu.cn; 3 Guangdong Provincial Key Laboratory of Aquatic Animal Disease Control and Healthy Culture, Zhanjiang 524088, China

**Keywords:** *Isochrysis galbana*, light-emitting diode (LED), light intensity, fucoxanthin, polyunsaturated fatty acids (PUFAs), co-production

## Abstract

This study assessed the effects of different intensities of broad-spectrum white LED light (PAR range: 415–748 nm) on growth, fucoxanthin accumulation, and fatty acid composition of *Isochrysis galbana*. This study classified light intensity into three categories based on the white LED light source: high (HL, 150 μmol·m^−2^·s^−1^), medium (ML, 80 μmol·m^−2^·s^−1^), and low (LL, 30 μmol·m^−2^·s^−1^). The results showed that biomass concentration was optimized under high light intensity (HL, 150 μmol·m^−2^·s^−1^), whereas low light (LL, 30 μmol·m^−2^·s^−1^) yielded the highest fucoxanthin concentration (71.15 mg/L on day 12) and the only positive volumetric fucoxanthin productivity (3.14 mg/L/d) among the three treatments tested. The results further showed that low light (LL, 30 μmol·m^−2^·s^−1^) produced maximum cell density (10.08 × 10^6^ cells/mL) and the most polyunsaturated fatty acids (PUFAs), particularly eicosapentaenoic acid (EPA) and docosahexaenoic acid (DHA), which constituted 1.93% and 22.47% of total fatty acids, respectively. This study demonstrates that low-intensity (LL, 30 μmol·m^−2^·s^−1^) white LED light supports the maximum co-production of valuable metabolites in *I. galbana*, establishing a scientific basis for scaling up *I. galbana* cultivation for nutraceutical and aquafeed applications.

## 1. Introduction

*Isochrysis galbana* is a unicellular marine haptophyte microalga within the order Isochrysidales, first described by Parke in 1949. It typically measures 2–5 μm in diameter and possesses a parietal chloroplast with organized thylakoids, and its golden-brown coloration reflects a pigment composition dominated by chlorophyll a, chlorophyll c, and fucoxanthin. Microalgae have emerged as a promising source for the sustainable production of high-value bioproducts, including carotenoids and polyunsaturated fatty acids (PUFAs), due to their rapid growth rates, metabolic flexibility, and ability to utilize CO_2_ efficiently [1,2,3]. Among marine microalgae, *I. galbana* is important because of its dual capacity to synthesize fucoxanthin and long-chain PUFAs such as eicosapentaenoic acid (EPA) and docosahexaenoic acid (DHA), both of which have significant commercial value in aquaculture, pharmaceutical, and nutraceutical industries [4,5,6,7,8,9,10].

Fucoxanthin is a major xanthophyll pigment in brown algae and certain microalgae, known for its strong antioxidant, anti-inflammatory, and anti-obesity properties [11]. Due to these bioactivities, fucoxanthin has gained increasing attention as a high-value compound for functional foods and therapeutic applications. At the same time, microalgae rich in PUFAs such as EPA and DHA are extensively added into aquafeed formulations as whole-cell biomass or lipid extracts, improving the omega-3 content and overall nutritional quality of farmed fish and shellfish [12,13,14]. Optimizing cultivation conditions for *I. galbana* therefore requires an integrated approach that accounts for both fucoxanthin and PUFA biosynthesis simultaneously, a strategy supported by recent modelling studies on light–temperature–nutrient interactions in this species [15]. Therefore, microalgae capable of producing both compounds simultaneously represent an attractive target for biotechnological exploitation.

Light intensity is one of the most critical environmental factors regulating microalgal physiology, as it directly affects photosynthetic performance, carbon assimilation, and metabolic flux distribution [16]. Previous studies have demonstrated that light intensity influences biomass accumulation, pigment synthesis, and lipid metabolism in *I. galbana* [17,18]. Recent studies on *I. galbana* and the genus *Isochrysis* have further elucidated the influence of light quality and intensity on fucoxanthin accumulation, lipid composition [19], and the co-production of high-value metabolites [20]. However, most existing studies have focused on optimizing a single metabolic output, such as fucoxanthin or lipid production, often under varying cultivation systems and light regimes.

Despite these advances, a key limitation remains in the lack of integrated studies examining the simultaneous regulation of multiple high-value metabolites. In particular, the relationship between fucoxanthin accumulation and fatty acid biosynthesis under controlled light intensities is still not fully understood. However, whether these metabolic pathways operate independently, competitively, or synergistically under different light conditions remains unresolved. This gap constrains the development of efficient cultivation strategies for maximizing overall productivity over that of a single compound, as improving growth and yield requires understanding how these metabolic pathways interact under varying light conditions. Although light-induced changes in metabolite accumulation have been widely reported [17], the physiological basis underlying these responses, such as energy partitioning, redox balance, and enzymatic regulation of lipid desaturation, has often been insufficiently discussed. A better understanding of these processes is essential for interpreting experimental results and improving process optimization.

Therefore, the present study systematically evaluated the effects of varying broad-spectrum white LED light intensities on growth, fucoxanthin accumulation, and fatty acid profiles of *I. galbana*. This study aimed to identify optimal light conditions for the co-production of fucoxanthin and PUFAs and provide a physiologically relevant interpretation of light-driven metabolic responses. This approach bridges the gap between fundamental micro-algal physiology and applied bioprocess optimization.

## 2. Materials and Methods

### 2.1. I. galbana Cultivation and Harvesting

The *I. galbana* was provided by the Algal Resource Development and Aquatic Environmental Ecological Restoration Laboratory at Guangdong Ocean University (Zhanjiang, China). The algal strain used in this study is not deposited in a public collection, and therefore has no accession number.

The algal strain was cultivated in a modified F/2 natural seawater medium (collected from the South China Sea, Zhanjiang), which contained the following components per liter: NaNO_3_ (75 mg), NaH_2_PO_4_·H_2_O (5 mg), Na_2_SiO_3_·9H_2_O (30 mg), and F/2 trace element solution (1 mL). The F/2 trace element solution included FeCl_3_·6H_2_O (3150 mg), Na_2_EDTA·2H_2_O (4360 mg), CuSO_4_·5H_2_O (9.8 mg), Na_2_MoO_4_·2H_2_O (6.3 mg), ZnSO_4_·7H_2_O (22 mg), CoCl_2_·6H_2_O (10 mg), and MnCl_2_·4H_2_O (180 mg). Culture conditions were maintained at a constant temperature of 25 °C, with a fluorescent light intensity of 30 ± 2 μmol·m^−2^·s^−1^ and continuous aeration.

When the strain was cultured to the exponential growth phase, it was centrifuged to a specific concentration for inoculation and experimentation.

### 2.2. Experiment Design

**Figure 1:**
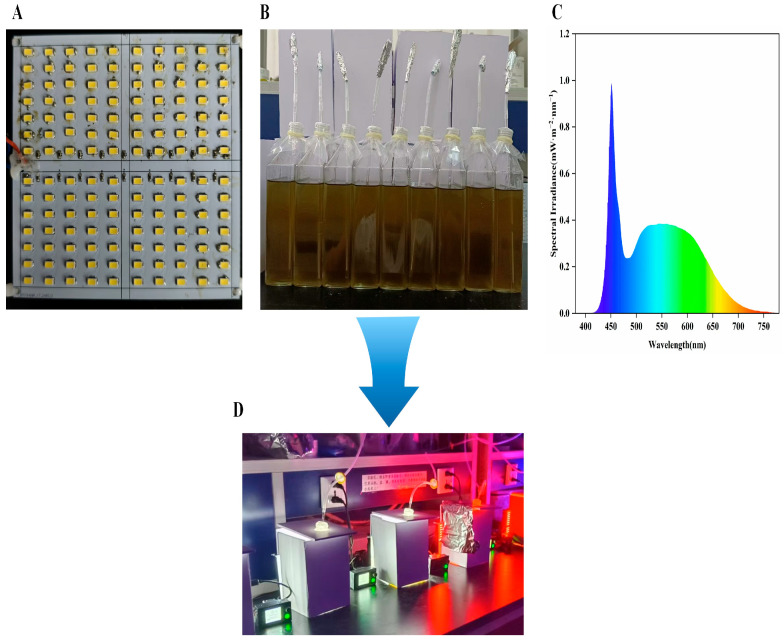
LED lighting setup and algae cultivation imagery. (**A**) Configuration of LED chips on the white LED light panel; (**B**) cultivation vessels and suspension aliquoting for *I. galbana*; (**C**) spectral schematic of white LED light; (**D**) integrated experimental apparatus.

In this study, a broad-spectrum white LED panel (PAR range: 415–748 nm) was employed as the light source (Figure 1A–D). The LED source covers only the photosynthetically active radiation (PAR) spectrum; it is not full-spectrum white light in the strict physical sense. The light source exhibited a peak wavelength at 451 nm and a dominant wavelength at 486 nm. As shown in Figure 1C, the LED output contained a substantially more pronounced blue-light fraction than that of natural sunlight, a characteristic of phosphor-converted white LEDs. The potential independent effects of spectral composition are discussed in Section 4. Different light-intensity gradients (150, 80, and 30 μmol·m^−2^·s^−1^) corresponding to high-intensity light (HL), medium-intensity light (ML), and low-intensity light (LL), respectively, were applied to *I. galbana* throughout its growth phase. This experiment included three biological replicates to ensure the reliability of the results.

The experiments were carried out in rectangular-necked cell culture flasks (110 mm in length, 50 mm in width, 200 mm in height, with a sidewall thickness of 3 mm and 800 mL capacity) (Figure 1B) with a culture volume of 600 mL. The algal inoculation density was about 3.65 × 10^6^ cells/mL. The culture conditions were as follows: continuous illumination, a temperature of 25 ± 2 °C (maintained by thermostat-regulated air conditioning), salinity of 25 ± 2, pH of 8.0 ± 0.2 (monitored daily using a calibrated pH meter; pH remained stable within this range due to the natural buffering capacity of the seawater-based medium and moderate CO_2_ supply from aeration, and no active CO_2_ injection or buffer addition was employed), and aeration at 0.4 L/min providing continuous mixing. The experimental period was 15 days, with sampling every 3 days to measure algal cell density, dry cell weight, and fucoxanthin content. After the cultivation period, the algae were collected by centrifugation and freeze-dried for fatty acid content and composition analysis.

### 2.3. Analytical Methods

Cell density was counted using a hemocytometer (25 × 16 mm) under a BX53 fluorescence microscope (Shimadzu Corporation, Tokyo, Japan) after staining the sample concentration cells with iodine solution.

Dry weights (DWs) were determined using the differential gravity method [21]. Aliquots of 10 mL algal suspension were filtered through pre-weighed (M_1_) and pre-dried (80 °C, overnight) microporous filter membrane (Mixed Cellulose Ester (Water-based), 47 mm, nominal pore size 1 μm; Shangahi Xingya Purification Material Factory, Shanghai, China), pre-washed five times using 10–15 mL of deionized water, and dried overnight in an 80 °C oven. The filters were subsequently cooled in a desiccator prior to weighing. Weights (M_2_) were measured on an analytical balance with a precision of 0.1 mg. The dry weights of the algal cells were calculated using Equation (1).

DW (g/L) = (M_2_ − M_1_) × 10^3^/V (1)

where M_1_ is the pre-dried filter weight (g), M_2_ is the post-drying filter + biomass weight (g), and V is the filtration volume (mL = 10).

#### 2.3.1. Fucoxanthin Extraction and Analysis

Fucoxanthin was extracted from 15 mg of freeze-dried algal cells using pure ethanol (10 mL) for 15 min. The mixture was subsequently centrifuged for 10 min at 10,000 rpm, and the supernatant was collected. The procedure was repeated twice, after which the combined supernatant was evaporated to dryness under a nitrogen stream and dissolved in 1 mL of ethanol for further analysis. All procedures were conducted in the dark to prevent fucoxanthin degradation [22]. The extracted fucoxanthin was quantified using an HPLC System Series 1000 (Agilent Technologies, Santa Clara, CA, USA) equipped with a UV detector. The analysis utilized a reverse-phase Acclaim 120 C18 column (150 × 4.6 mm, 5 μm; Thermo Fisher Scientific, Waltham, MA, USA) maintained at 30 °C. A sample volume of 10 μL was injected into the column and eluted with a water–acetonitrile mixture (1:3). The isocratic elution program was executed at a detection wavelength of 450 nm, flow rate of 1.8 mL/min, and duration of 30 min. A standard curve was established using standard products (Sigma-Aldrich, St. Louis, MO, USA) ranging from 0.5 to 20 mg/L.

Fucoxanthin productivity (FP, mg^−1^ d^−1)^ was calculated using the following equation:

FP = (C_t_ − C_0_)/t (2)

where C_t_ and C_0_ are the fucoxanthin contents on day t and day 0, respectively.

#### 2.3.2. FAME Analyses

For fatty acid methyl ester (FAME) analyses, 300 mL samples from each treatment were collected, centrifuged at 8000× *g* for 5 min following incubation, and freeze-dried for 30 min using an Alpha 1–2 LD plus (Martin Christ Gefriertrocknungsanlagen GmbH, Osterode am Harz, Germany). The biomass was hydrolyzed and methyl-esterified in 300 μL of a 2% (*v*/*v*) H_2_SO_4_ methanol solution at 80 °C for 2 h. Then, 50 μg of C21:0 (Heneicosanoic acid, Sigma-Aldrich) was added as an internal recovery standard. After esterification, 300 μL of 0.9% (*w*/*v*) NaCl solution and 300 μL of n-hexane were added to the samples, followed by vortexing for 20 s and centrifugation at 16,000× *g* for 3 min to separate the phases. Subsequently, 150 μL of the n-hexane layer was analyzed using an Agilent 6890 gas chromatography (Agilent 6890; Agilent Technologies, Santa Clara, CA, USA) coupled with a 5975 MSD mass spectrometer (Agilent Technologies, Santa Clara, CA, USA), following Agilent’s RTL DBWax method (5988-5871EN) [23]. Fatty acids were identified by comparing their retention times with those of pre-run external standards (37 FAME Mix, Supelco, Supelco, Bellefonte, PA, USA) using the NIST library. Fatty acid components were quantified using the area normalization method, and the total fatty acid (TFA) content was determined by summing all identified fatty acids.

#### 2.3.3. Statistical Analysis

We calculated the mean and standard deviation for all treatments. SPSS Statistics software (version 23) was used to perform a one-way repeated-measures ANOVA, and pairwise comparisons were used to determine significant (*p *< 0.05) differences between treatments for growth, carotenoids, and fucoxanthin content. One-way ANOVA with Duncan’s test (post hoc) was used to determine significant (*p *< 0.05) differences between treatments for the fatty acid profile.

## 3. Results

### 3.1. Growth of I. galbana Under White LED Light Intensities

**Figure 2:**
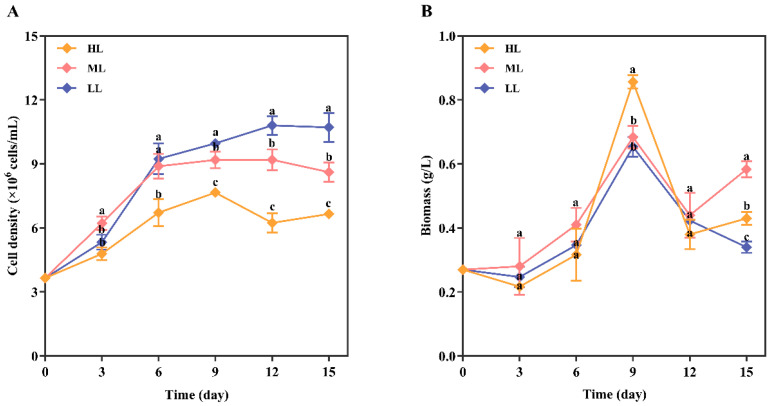
*I. galbana* cell density and biomass changes under white LED light intensities. (**A**) Changes in cell density of *I. galbana* under HL, ML, and LL conditions over time (0–15 days), with data presented as mean ± SD (*n* = 3); (**B**) dynamic changes in biomass under the corresponding conditions. The letters a, b, and c in the figure are used to indicate the differences among the treatment groups. Groups marked with the same letter are not significantly different, while those with different letters show significant differences (*p* < 0.05).

Over the cultivation period, the cell densities in all three groups increased from day 0 to 9, with the HL group peaking at 7.66 × 10^6^ cells/mL on day 9 before declining. On day 12, the LL group attained a peak cell density of 10.8 × 10^6^ cells/mL, which was significantly higher than that of the ML group (9.19 × 10^6^ cells/mL; *p* < 0.05) and other groups, before both groups subsequently declined (Figure 2A).

Cell biomass in the ML group increased steadily, peaking at 0.92 g/L on day 9. In contrast, the HL and LL groups exhibited a transient decline in dry weight on day 3, followed by continuous growth until day 9 (reaching 0.86 g/L and 0.65 g/L, respectively). Throughout the cultivation period, the HL group showed significantly higher biomass accumulation than other groups (*p* < 0.05) (Figure 2B). The result showed that cell density and dry biomass did not exhibit the same trend across treatments, likely because light conditions altered cell size and intracellular composition, including lipid and pigment content, resulting in a decoupling between cell number and total biomass [18,19].

### 3.2. Fucoxanthin Content in I. galbana Under White LED Light Intensities

**Figure 3:**
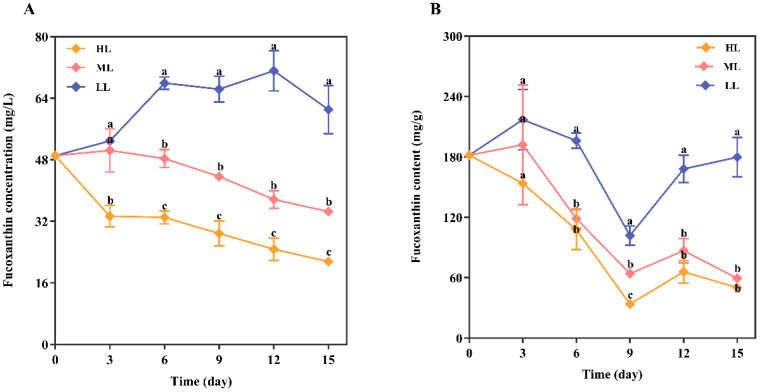
Changes in fucoxanthin concentration and content of *I. galbana* under white LED light intensities. (**A**) Changes in fucoxanthin concentration of *I. galbana* under HL, ML, and LL conditions over time (0–15 days), with data presented as mean ± SD *(n* = 3); (**B**) changes in fucoxanthin content under the corresponding conditions. The letters a, b, and c in the figure are used to indicate the differences among the treatment groups. Groups marked with the same letter are not significantly different, while those with different letters show significant differences (*p* < 0.05).

The results showed that different white-light intensities affected the fucoxanthin concentration in *I. galbana*. The HL and ML groups showed a gradual decline from the start, indicating an inhibitory effect. In contrast, the LL group gradually increased, peaking at 71.15 mg/L on day 12 before declining (Figure 3A).

The HL group inhibited fucoxanthin accumulation in *I. galbana*, with levels declining throughout the experiment and slightly increasing on day 12 before dropping again. The ML group reached a maximum on day 3 (192.05 mg/g) and then declined thereafter. The LL group promoted fucoxanthin accumulation, peaking on day 3 (217.12 mg/g), dropping sharply afterward, and then increasing from day 12 to the end of the experiment (Figure 3B).

### 3.3. I. galbana Productivity Under White LED Light Intensities

**Figure 4:**
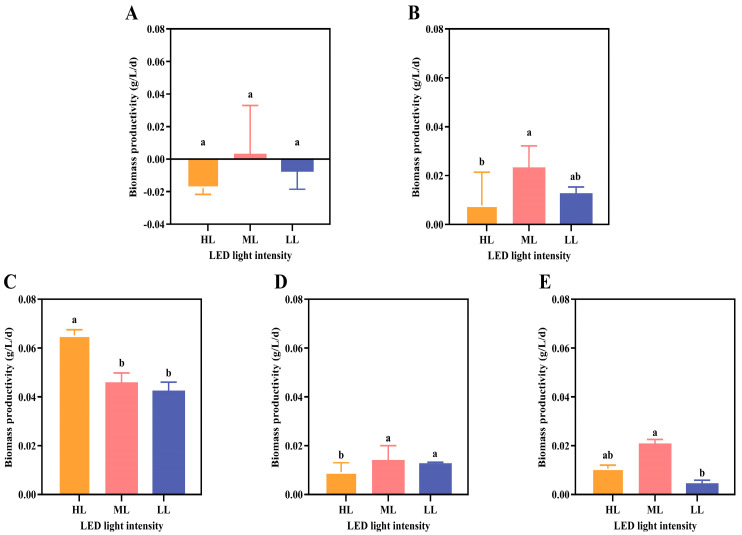
Biomass productivity of *I. galbana* under white LED light intensity. (**A**–**E**) on days 3 (**A**), 6 (**B**), 9 (**C**), 12 (**D**), and 15 (**E**) under HL, ML, and LL conditions, with data presented as mean ± SD (*n* = 3). The letters a and b in the figure are used to indicate the differences among the treatment groups. Groups marked with the same letter are not significantly different, while those with different letters show significant differences (*p* < 0.05).

**Figure 5:**
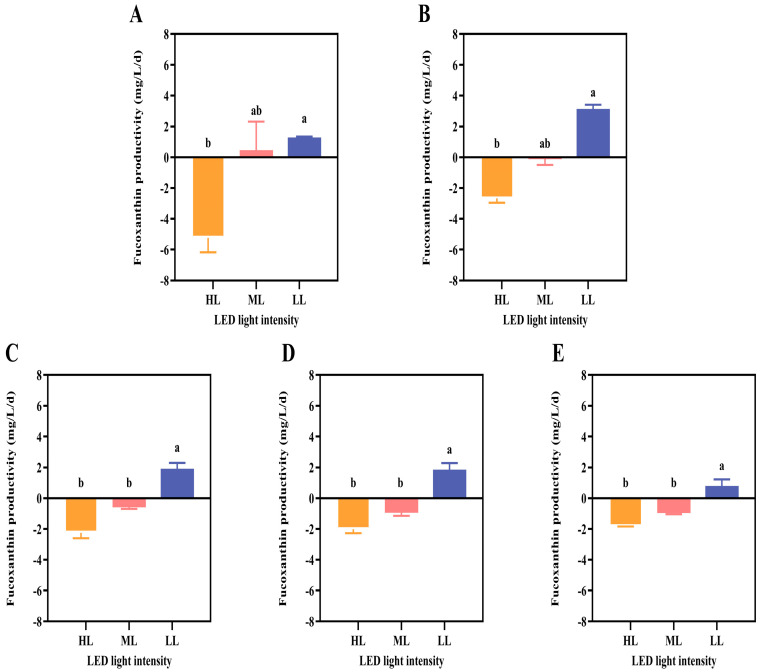
Fucoxanthin productivity of *I. galbana* under white LED light intensities. (**A**–**E**) on days 3 (**A**), 6 (**B**), 9 (**C**), 12 (**D**), and 15 (**E**) under HL, ML, and LL conditions, with data presented as mean ± SD (*n* = 3). The letters a and b in the figure are used to indicate the differences among the treatment groups. Groups marked with the same letter are not significantly different, while those with different letters show significant differences (*p* < 0.05). Note: Negative productivity values indicate that the net rate of fucoxanthin loss from the culture through photodegradation and dilution by cell division exceeded de novo biosynthesis during that interval, resulting in a net reduction in fucoxanthin concentration.

We compared biomass and fucoxanthin productivity under different light intensities on days 3, 6, 9, 12, and 15. In terms of biomass productivity, the HL group reached a maximum of 0.065 g/L/day on day 9, whereas the ML and LL groups experienced lower biomass productivity (Figure 4A–E). In terms of fucoxanthin productivity, the LL group consistently showed higher values, reaching a maximum productivity of 3.14 mg/L/day on day 6. Except on day 3, the HL and ML groups showed consistent negative fucoxanthin productivity values, indicating that the rate of fucoxanthin degradation and dilution by biomass growth exceeded de novo biosynthesis during those intervals, resulting in a net reduction in fucoxanthin concentration (Figure 5A–E).

### 3.4. Fatty Acid Profile of I. galbana Under White LED Light Intensities

At the end of the cultivation period, 26 fatty acids were detected in *I. galbana* under different white LED light intensities, including 14 saturated fatty acids (SFAs) and 12 unsaturated fatty acids (UFAs). Comparative analysis revealed that within each treatment group, the total unsaturated fatty acid (UFA) content exceeded that of the total saturated fatty acid (SFA) content. This variation was particularly driven by higher levels of specific UFAs (C18:1 and C22:6) and SFAs (C14:0 and C16:0) across all treatments. The fatty acids were present in the following order of abundance: SFAs (35.9–44.0%), MUFAs (28.8–31.8%), and PUFAs (24.2–35.3%). The dominant fatty acids were C14:0, C16:0, C18:1, and C22:6 (DHA), which is consistent with the typical lipid profile described for *I. galbana* across multiple strains [19,24,25]. HL treatment favored accumulation of C11:0, C14:0, C16:0, C18:0, C18:1, C18:2, C21:0, and C23:0. ML was conducive to C10:0, C20:0, C20:3n3, C20:5 (EPA), C22:0, C22:1, C22:2, C22:6 (DHA), and C24:0. LL promoted C12:0, C13:0, C14:1, C15:0, C16:1, C17:0, C18:3n6, and C18:3n3 (α-linolenic acid, ALA) (Table 1).

## 4. Discussion

The interaction between the light spectrum and light intensity is complex, and the appropriate combination is a key factor in regulating microalgal growth, photosynthesis, and biomass accumulation [26,27]. A previous study revealed that under optimal nitrate concentrations, *Pavlova lutheri*, *Chlorella vulgaris*, and *Porphyridium cruentum* obtained maximum biomass under 100 μmol·m^−2^·s^−1^ LED wavelengths [28]. *Isochrys galbana* has been identified as a species of biotechnological interest due to its capacity to accumulate fucoxanthin and long-chain PUFAs [19,20]. The present study demonstrates that light intensity significantly affects the growth, fucoxanthin accumulation, and fatty acid profiles of *I. galbana*, with distinct optima for biomass and metabolite quality. It should be noted that the broad-spectrum white LED panel used in this study exhibited a pronounced blue-light peak at 451 nm (Figure 1C), with blue wavelengths (400–500 nm) comprising a disproportionately large fraction of the total photon output relative to natural sunlight. Blue light is known to independently affect fucoxanthin biosynthesis, fatty acid desaturation, and growth in *I. galbana* [29]. Accordingly, the biological responses observed in this study likely reflect the combined effects of total irradiance and spectral quality, rather than photon flux density alone. Future studies employing spectrally matched or monochromatic light sources would help disentangle the independent contributions of irradiance intensity and blue-light proportion.

Our study demonstrates that light intensity affects growth, fucoxanthin, and fatty acid accumulation in *I. galbana.* The findings of the present study showed variations in cell density and dry biomass weight among the different treatments (Figure 2). Maximum cell density was observed at low light (30 μmol·m^−2^·s^−1^) intensity, whereas maximum dry weight was observed at medium light (80 μmol·m^−2^·s^−1^) intensity. The peak biomass dry weight of 0.92 g/L observed under ML (80 μmol·m^−2^·s^−1^) suggests that this irradiance represents the photosynthetic optimum for this strain, providing sufficient photon flux to support maximum carbon fixation without triggering photoprotective energy-dissipation mechanisms that can reduce growth efficiency under HL. The lower biomass observed under LL likely reflects the limitation of photosynthetic electron transport due to insufficient photon supply, whereas the HL-induced photoinhibition, evidenced by the transient biomass decline on day 3, likely resulted from temporary photosystem II damage that reduced net carbon assimilation [30]. The highest biomass under medium light intensity suggests that medium light levels provide an optimal balance between light absorption and photochemical utilization [31,32]. The maximum biomass observed at medium light intensity is consistent with previously reported values in another study, where *I. galbana* obtained a maximum biomass of 1.04 g dcw/L under 400 μmol·m^−2^·s^−1^ light intensity [33].

Light-induced variation in fucoxanthin production in *I. galbana* was evident in each of the three treatments evaluated. Fucoxanthin concentration in *I. galbana* from the low-intensity light (LL) group were found to progressively accumulate over time with the highest concentration recorded on day 12 at 71.15 mg/L with maximum productivity of 3.14 mg/L/d). In contrast, both the high-intensity light (HL) and medium-intensity light (ML) groups showed consistent declines throughout the cultivation period (Figure 3A,B).

As described in Section 2.1, the inoculum was maintained under the same low-light conditions (30 μmol·m^−2^·s^−1^ fluorescent light) used for the LL treatment. Consequently, the day 0 fucoxanthin concentration reflected a pre-acclimated, low-light physiological state rather than a neutral baseline. When transferred to HL or ML conditions, cultures experienced a sharp and sustained decline in both fucoxanthin concentration and fucoxanthin content per unit biomass, confirming the inhibitory effect of higher irradiance on fucoxanthin biosynthesis in *I. galbana*. In contrast, the LL group maintained and ultimately recovered its fucoxanthin concentration, peaking at 71.15 mg/L on day 12, approximately 45% above the day 6 trough, despite a three- to fourfold increase in biomass across all groups. This demonstrates that LL cultures were capable of sustaining de novo fucoxanthin biosynthesis at a rate sufficient to support both cellular demand and increased culture volume, a capacity entirely absent in the HL and ML groups. The positive fucoxanthin productivity observed exclusively in the LL group (3.14 mg/L/day on day 6) reflects a genuine biosynthetic advantage under low irradiance, not merely the retention of pre-acclimation conditions.

Under photon-limited conditions, *I. galbana* upregulates fucoxanthin–chlorophyll a/c protein (FCP) complexes to increase the effective antenna size and maximize light energy capture [34,35]. Conversely, under HL, reactive oxygen species (ROS) generated by excess photon flux induce oxidative damage to both the FCP apparatus and fucoxanthin biosynthetic capacity, consistent with the pigment degradation observed in our HL treatment [36]. The decline in fucoxanthin content per unit biomass (mg/g DW) under ML, despite steady dry weight accumulation, further suggests that pigment dilution by rapid cell division outpaced biosynthesis at moderate irradiance. These results are consistent with those of Bo et al. [33] and Maduraimuthu et al. [37], who identified 30–50 μmol·m^−2^·s^−1^ as the optimal light intensity for fucoxanthin accumulation in *I. galbana*, and confirmed that 30 μmol·m^−2^·s^−1^ broad-spectrum white LED intensity drives fucoxanthin productivity through adaptive antenna expansion, rather than constitutive synthesis.

Fatty acid analysis of *I. galbana* revealed 26 fatty acids across the three treatments, with C14:0, C16:0, C18:1, and DHA (C22:6n-3) as the dominant components, a profile consistent with the characteristic lipid profile of this species reported for several strains [19,24,25]. The predominance of DHA among the PUFAs further supports the characterization of *I. galbana* as a DHA-rich microalga.

The LL treatment yielded the highest PUFA proportion (35.3% of total FA), with EPA and DHA accounting for 1.93% and 22.47% of total FA, respectively. ML treatment produced the highest absolute DHA concentration (21,361 μg/g), whereas HL resulted in the lowest PUFA proportion (24.2%) and DHA content (15,981 μg/g). These gradients demonstrate inverse relationship between light intensity and degree of polyunsaturation, consistent with light-dependent regulation of fatty acid desaturase activity. Wu et al. [24] observed similar high DHA content in *I. galbana* under conditions that promote PUFA biosynthesis, and Putra et al. [25] reported DHA as the dominant PUFA in *I. galbana* clone t-ISO. Meneses-Montero et al. [19] established that PUFAs typically exceed 15% of total FA in productive *I. galbana* strains, a threshold exceeded in both the LL (35.3%) and ML (30.0%) groups in the present study. This supports the biotechnological quality of the accumulated biomass.

Studies on *I. galbana* support and extend these findings. Aguilera-Sáez et al. [18] showed that increasing irradiance shifts metabolism from polar unsaturated lipids to neutral saturated fatty acids, which is consistent with the observed decline in PUFA from low-intensity to high-intensity light. Mishra et al. [17] also reported that high irradiance combined with nitrate limitation reduces chlorophyll and carotenoid content while promoting saturated fatty acid accumulation, while DHA is uniquely maintained, suggesting partial decoupling of DHA synthesis from total PUFA regulation. Jin et al. [29] further demonstrated that light quality modulates photosynthesis and bioactive compound production, indicating that the blue-enriched LED spectrum (451 nm) likely influenced the responses observed. Collectively, these studies indicate that low irradiance favors fucoxanthin and PUFA accumulation, whereas higher irradiance redirects metabolism toward biomass and saturated lipids, supporting the broader applicability of these findings for optimizing bioprocesses.

The LL treatment promoted accumulation of ALA (C18:3n-3), which serves as a precursor to longer-chain omega-3 PUFAs, including EPA and DHA, through the action of elongases and desaturases [19]. Conversely, HL promoted C18:1 (oleic acid), which is characteristic of a more saturated lipid profile consistent with photoprotective storage lipid accumulation under high irradiance. Fatty acid desaturation activity depends on the supply of NADPH from photosynthetic light reactions [38]; therefore, changes in light intensity alter the intracellular redox balance, influencing desaturase enzyme activity and the degree of lipid unsaturation.

Under low light, the simultaneous enhancement of fucoxanthin productivity and PUFA proportion in *I. galbana*, rather than a strict metabolic trade-off, may reflect an adaptive strategy in which this species expands both its light-harvesting capacity through FCP complexes and its membrane plasticity through PUFA enrichment to improve photosynthetic performance under energy-limited conditions [35,38]. These results support the co-production of fucoxanthin and PUFAs at 30 μmol·m^−2^·s^−1^, strengthening the economic and environmental rationale for this light regime in industrial bioprocessing. Further studies using transcriptomic or metabolomic profiling will clarify the molecular mechanisms underlying light-driven lipid remodeling in *I. galbana*.

## 5. Conclusions

This study systematically assessed the influence of varying intensities of broad-spectrum white LED light on the growth, fucoxanthin accumulation, and fatty acid profiles of *I. galbana* during a 15-day cultivation period. Light intensity has distinct effects on biomass accumulation and metabolite biosynthesis, and no single intensity optimizes all outputs simultaneously. High light intensity (HL, 150 μmol·m^−2^·s^−1^) promoted dry biomass accumulation (0.86 g/L on day 9) but suppressed fucoxanthin biosynthesis and decreased the proportion of polyunsaturated fatty acids (PUFAs, 24.2% of total fatty acids). Medium light intensity (ML, 80 μmol·m^−2^·s^−1^) produced the maximum dry biomass (0.92 g/L) and the highest absolute DHA concentration (21,361 μg/g), indicating an optimal irradiance level for carbon fixation and DHA accumulation in this study. Low light intensity (LL, 30 μmol·m^−2^·s^−1^) yielded the maximum cell density (1.08 × 10^7^ cells/mL on day 12); highest PUFA proportion (35.3% of total FA), with EPA at 1.93% and DHA at 22.47%; and the only sustained positive fucoxanthin productivity among the three treatments (3.14 mg/L/day), with a peak fucoxanthin concentration of 71.15 mg/L.

The concurrent increase in fucoxanthin productivity and PUFA accumulation under LL conditions aligns with a coordinated adaptive response in *I. galbana*, in which energy-limited conditions promote the simultaneous expansion of light-harvesting fucoxanthin–chlorophyll protein (FCP) complexes and enrichment of membrane polyunsaturated fatty acids to maintain photosynthetic efficiency. These findings support the co-production of fucoxanthin and omega-3 PUFAs under a single low-irradiance regime, with the DHA proportion (22.47% of total FA) meeting quality benchmarks for aquafeed supplementation and reducing the need for sequential metabolite-specific cultivation strategies.

Future studies should employ spectrally controlled light sources and integrate transcriptomic profiling to resolve the molecular mechanisms underlying light-driven metabolite co-regulation in *I. galbana*, and photobioreactor scale-up trials will be required to confirm commercial feasibility.

## Figures and Tables

**Table 1:** Fatty acid composition and concentrations (μg/g) of *I. galbana* under white LED light.

Test Items	White LED Light Intensities		
	HL (150 µmol·m^−2^·s^−1^)	ML (80 µmol·m^−2^·s^−1^)	LL (30 µmol·m^−2^·s^−1^)
C10:0	302.55	757.18	406.77
C11:0	95.71	67.10	73.28
C12:0	Not detected	47.58	64.41
C13:0	Not detected	Not detected	49.31
C14:0	14,665.00	12,707.01	12,057.35
C14:1	Not detected	71.87	86.57
C15:0	524.90	617.56	751.08
C16:0	28,659.73	23,250.46	14,755.10
C16:1	3111.85	3233.03	3871.05
C17:0	216.85	282.40	324.62
C18:0	1387.25	1378.82	951.03
C18:1	29,531.55	25,344.44	18,504.28
C18:2	3319.42	3018.63	2732.69
C18:3n6	Not detected	52.53	112.47
C18:3n3	4989.96	5687.04	6026.39
C20:0	270.24	304.46	260.71
C20:1	Not detected	93.39	69.11
C20:3n3	295.47	421.88	383.06
EPA	1515.73	1666.57	1663.40
C21:0	133.04	126.13	113.33
C22:0	1333.86	1422.08	999.76
C22:1	1883.46	2558.57	2266.72
C22:2	137.38	199.56	160.72
DHA	15,980.69	21,361.22	19,369.02
C23:0	159.32	142.86	138.96
C24:0	Not detected	3224.88	Not detected
SFA	47,748.44	44,328.53	30,945.71
UFA	60,765.50	63,708.74	55,245.49
MUFA	34,526.86	31,301.31	24,797.73
PUFA	26,238.64	32,407.43	30,447.75
SFA (%)	44.0	41.0	35.9
UFA (%)	56.0	59.0	64.1
MUFA (%)	31.8	29.0	28.8
PUFA (%)	24.2	30.0	35.3
FA	108,513.94	108,037.27	86,191.20

Note: SFA: saturated fatty acid; UFA: unsaturated fatty acid; MUFA: monounsaturated fatty acid; PUFA: polyunsaturated fatty acid; FA: total fatty acid.

## Data Availability

The original data presented in this study are included in the article; further inquiries can be directed to the corresponding author.
